# Multicentre Comparison of the Toxicity and Effectiveness of Lobaplatin-Based *Versus* Cisplatin-Based Adjuvant Chemotherapy in Oesophageal Carcinoma

**DOI:** 10.3389/fonc.2021.668140

**Published:** 2021-09-13

**Authors:** Yan Zheng, Yin Li, Xianben Liu, Haibo Sun, Guanghui Liang, Jiajia Hu, Liping Li, Wenqun Xing

**Affiliations:** ^1^Department of Thoracic Surgery, The Affiliated Cancer Hospital of Zhengzhou University, Henan Cancer Hospital, Zhengzhou, China; ^2^Department of Thoracic Surgery, National Cancer Center/National Clinical Research Center for Cancer/Cancer Hospital, Chinese Academy of Medical Sciences and Peking Union Medical College, Beijing, China; ^3^Department of Statistics, LinkDoc Technology Co., Ltd., Beijing, China

**Keywords:** ESCC, adjuvant chemotherapy, lobaplatin, adverse reactions, cisplatin

## Abstract

**Objectives:**

Lobaplatin (LBP), a third-generation cisplatin derivative has shown promising activity and few side effects in oesophageal squamous cell carcinoma (ESCC) in previous reports. We compared LBP plus docetaxel with cisplatin plus docetaxel as adjuvant chemotherapy in ESCC patients to determine the effects on overall survival (OS) and toxicity.

**Methods:**

A multicentre retrospective study was performed using propensity score matching (PSM) with the Medicine-LinkDoc database. Patients diagnosed with stage II-III ESCC treated with adjuvant chemotherapy (cisplatin plus docetaxel or LBP plus docetaxel) between January 2013 and December 2016 were selected from 6 centres in China.

**Results:**

There were 733 eligible ESCC patients. After PSM (1:1 ratio), 458 patients remained. The 5-year OS rates of the cisplatin and LBP groups were 25.9% and 23.6%, respectively (P=0.457). Leukopenia (grade III-IV/I-II/0: 2.62%/34.5%/59.39% *versus* 5.24%/43.23%/45.85%; P=0.0176), neutropenia (grade III-IV/I-II/0: 6.55%/37.56%/51.09% *versus* 4.37%/53.28%/36.34%; P=0.0015), nephrotoxicity (grade I-II/0: 13.97%/76.86% *versus* 26.64%/65.94%; P<0.001) and gastrointestinal symptoms (grade III-IV/I-II/0: 2.18%/54.59%/32.31% *versus* 6.55%/65.07%/20.88%; P=0.0011) were more frequent in the cisplatin group.

**Conclusions:**

Compared with cisplatin plus docetaxel, LBP plus docetaxel provided the same survival benefits but lower side effects of myelosuppression and gastrointestinal symptoms. LBP plus docetaxel might be a choice for adjuvant chemotherapy in ESCC.

**Clinical Trial Registration:**

Lobaplatin or Cisplatin in Adjuvant Chemotherapy for Oesophageal Carcinoma, identifier NCT03413436.

## Introduction

Over the past two decades, the combination of preoperative chemotherapy and chemoradiotherapy has become the standard of care for the systemic therapy of oesophageal squamous cell carcinoma (ESCC) in Western countries and Japan. In China, where more than half of the ESCC cases in the world occur, adjuvant chemotherapy (AC) or chemoradiotherapy has mainly been adopted (1). Because of postoperative complications and nutrition problems, AC in Western countries has rarely been administered. Lobaplatin (LBP), which has few side effects, has been adopted in AC to reduce side effects and increase the complete rate. Cisplatin-based regimens have been widely accepted as standard chemotherapy regimens worldwide and remain the standard of care for ESCC in China. However, the LBP regimen with less toxicity has subsequently emerged for older patients and is being evaluated for patients with low performance scores (PSs) (2).

The use of first- and second-generation platinum drugs such as cisplatin, carboplatin, and nedaplatin is often associated with drug resistance, nephrotoxicity, and bone marrow suppression. How to reduce chemotherapy-related toxicity without reducing the antitumour effect is an urgent problem to be solved. LBP, as a third-generation platinum compound, is basically similar to cisplatin in terms of DNA damage and cell apoptosis and does not need to be hydrated (3). LBP has played a reliable antitumour role in solid tumours such as lung cancer, nasopharyngeal cancer, breast cancer and gastric cancer (4–7). In *in vivo* animal experiments of ESCC, LBP has been shown to induce apoptosis and significantly inhibit the growth of ESCC. In the first-line treatment of patients with advanced ESCC, LBP has been shown to have certain efficacy and safety (8). However, due to the small sample sizes and lack of controlled trials, the existing studies cannot reflect the efficacy and safety advantages of LBP compared with chemotherapy regimens containing cisplatin. Therefore, our team conducted a retrospective study to understand AC combined with LBP after radical resection for ESCC in China and the difference in efficacy and safety between LBP and cisplatin. In addition, we aimed to understand the distribution characteristics of chemotherapy regimens and the characteristics of ESCC patients after treatment with radical resection combined with LBP AC.

The Medicine-LinkDoc database network provides a multicentre database of this topic for observational comparative-effectiveness studies of ESCC. We sought to compare the completion rates, toxicities and survival outcomes of ESCC patients receiving cisplatin- and LBP-based regimens as AC in real-world settings, employing propensity-matching methods to mitigate selection bias.

## Materials and Methods

**Figure 1:**
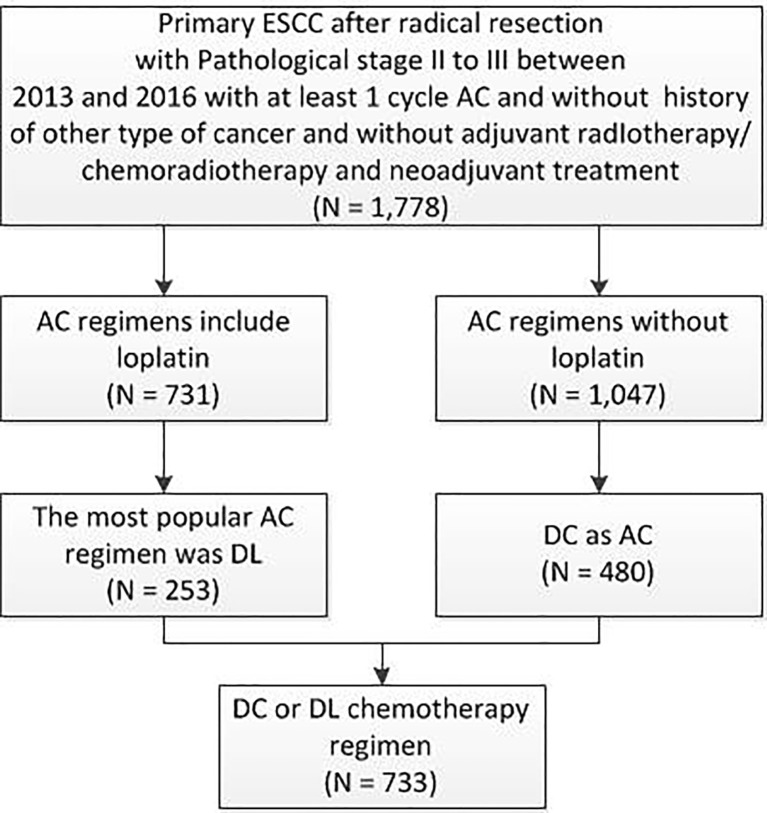
Patient distribution diagram. ESCC, oesophageal squamous cell carcinoma; AC, adjuvant chemotherapy; DL, docetaxel+ lobaplatin; DC, docetaxel+ cisplatin; N, number.

This study was approved by the ethics review committee of the Affiliated Cancer Hospital of ZhengZhou University/Henan Cancer Hospital and approved officially with approval number 2017405. Data from The Affiliated Cancer Hospital of Zhengzhou University/Henan Cancer Hospital, Anyang Cancer Hospital, Anhui Provincial Hospital, The First Affiliated Hospital of Anhui Medical University, Tangdu Hospital of the Fourth Military Medical University, and The First Affiliated Hospital of Xi’an Jiaotong University were combined to perform this retrospective study by using the Medicine-LinkDoc database network. A retrospective analysis was performed on patients with ESCC from the 6 centres who underwent radical resection from January 2013 to December 2016 (**Figure 1**). The inclusion criteria were as follows: pathological diagnosis of ESCC stage II/III, no surgical contraindications found, radical resection of ESCC performed as the primary treatment, at least 1 cycle of postoperative AC, and no radiotherapy performed in the same period. The exclusion criteria were as follows: history of other malignancies, preoperative treatment, and history of chemotherapy. The clinical data of the patients included the following: date of admission, sex, age, body mass index (BMI), past history, laboratory examination, clinical stage, tumour site, tumour size, degree of differentiation, lymph node metastasis, surgical history, number of chemotherapy cycles, etc.

**Figure 2:**
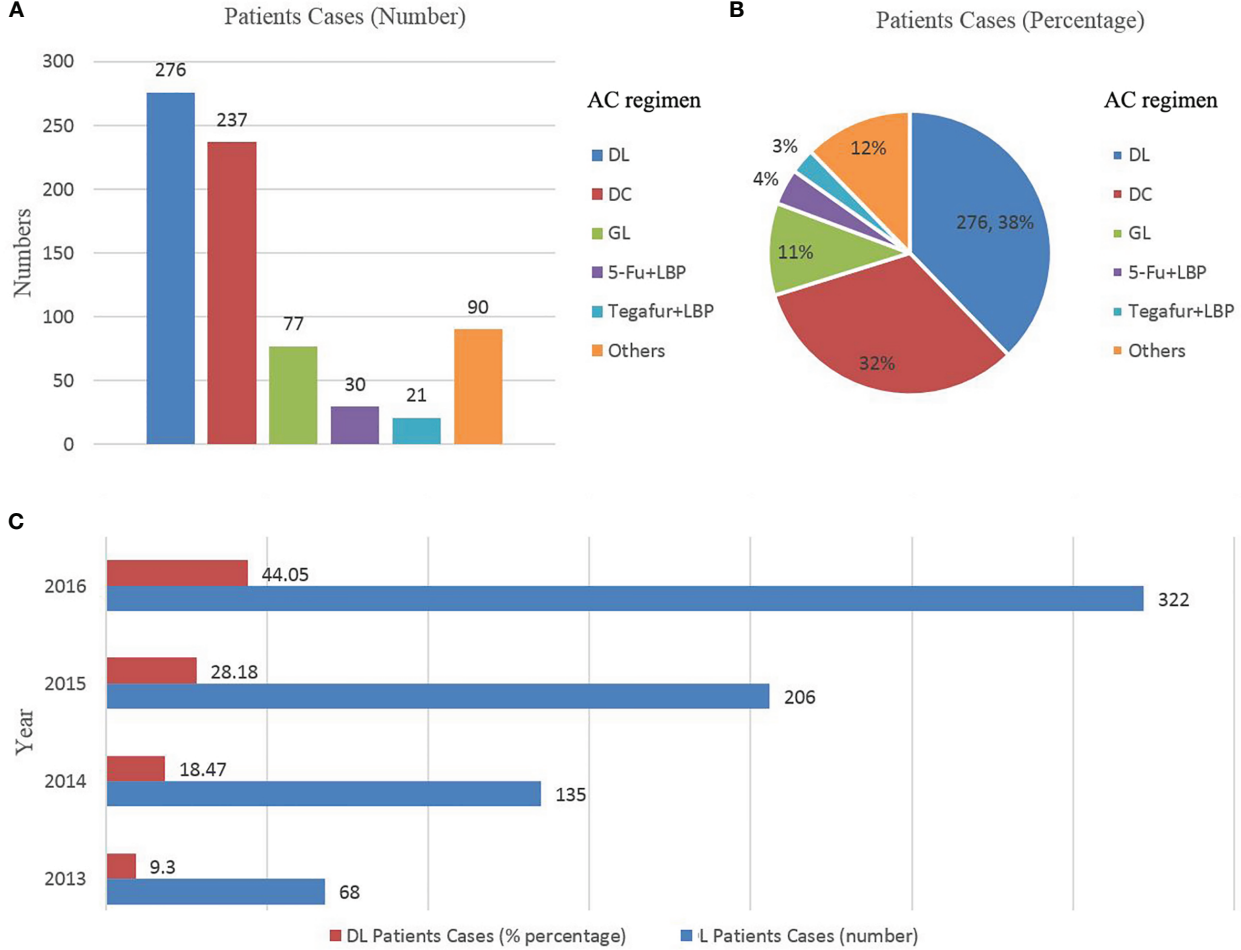
**(A, B)**, Scheme distribution of the combined lobaplatin regimens (N = 731); **(C)** Trends in the use of lobaplatin regimens by year, ESCC patients treated with a combined lobaplatin regimen from 2013 to 2016 and its percentage in all ESCC patients after AC (N = 731). DL, docetaxel+ lobaplatin; DC, docetaxel+ cisplatin; GL, gemcitabine + lobaplatin; LBP, lobaplatin; LBP, lobaplatin; N, number; AC, adjuvant chemotherapy; ESCC, oesophageal squamous cell carcinoma.

In the full cohort, the frequency distribution of chemotherapy regimens containing LBP was calculated (**Figures 2A, B**), in which the docetaxel combined with cisplatin regimen (docetaxel+ cisplatin, DC) had the highest frequency (276 cases, 37.76%), followed by paclitaxel combined with LBP (237 cases, 32.42%). Therefore, the docetaxel+LBP (DL) regimen was selected as the test group, and the DC regimen was selected as the control group (**Figure 1**). The dosages were usually docetaxel, 75–80 mg/m^2^ and cisplatin, 75 mg/m^2^; in the DL regimen, the dosages were docetaxel, 75–80 mg/m^2^ and lobaplatin, 50 mg/m^2^. Usually, 4 rounds of postoperative adjuvant chemotherapy are recommended. Total and subtotal thoracic oesophagectomies were performed. Right, left thoracotomy and thoracoscopic oesophagectomy were included. The transhiatal oesophagectomy was not used. Regional lymph nodes included mediastinal lymph nodes (paraesophageal, paratracheal, subcarinal, supradiaphragmatic and posterior mediastinal) and perigastric nodes. Bilateral recurrent laryngeal nerve lymph nodes were dissected if the right-side approach was adopted. Dissection of distant lymph nodes such as cervical nodes was reported in the cervical ultrasound test.

### Chemotherapy-Related Toxicities

The inpatient claims were all evaluated during the AC period. Chemotherapy-related toxicities were based on the World Health Organization (WHO) grading; bone marrow suppression, gastrointestinal side effects, liver and kidney function disorders, and electrocardiogram (ECG) changes were mainly evaluated during chemotherapy. Routine blood test results (white blood cells, platelets, lymphocytes and neutrophils), liver and kidney function test results (alanine aminotransferase, aspartate aminotransferase, creatinine, etc.) and ECG results were based on the WHO standards for severity classification of the outcome measure.

Any conditions before AC (heart failure, cerebrovascular accident, liver or kidney failure) were not included in the toxicity evaluation. The toxicity records were collected in the hospital. The model was performed with propensity matching and was adjusted for sex, tumour differentiation, pathological lymph node metastases, number of cycles of AC, and age.

### Survival

We measured overall survival (OS) as the days from the date of the operation to the date of death from any cause.

### Propensity Score Matching

Propensity score matching (PSM) is widely used to reduce selection bias in observational studies (9). The PSM method was used to match the two groups with a ratio of the test group to the control group of 1:1, considering the bias caused by confounding factors. The matching variables were based on clinical and methodological considerations, including sex, degree of differentiation, lymph node metastasis and the cycles of AC. We used PSM to create comparable cohorts of resected ESCC patients receiving DL and DC regimens on the basis of clinical and pathological characteristics.

### Analysis

Descriptive statistical methods were used to assess the baseline characteristics of the patients, and SAS 9.4 software was used for statistical analysis of the data. For continuous indicators, t-test or the Wilcoxon rank sum test was used for comparisons between two groups, and the chi-square test was used for classification data comparisons. For adverse events, the severity was graded, and the number and percentage of adverse events with different grades were obtained. Two-sided tests were used for all statistical tests, and P < 0.05 was considered statistically significant. We compared the Kaplan-Meier curves of the 5-year survival rates for DL and DC in the matched cohort. The Kaplan-Meier method was used to draw survival curves, and the log-rank test was used to compare the survival curves.

## Results

### Predictors of Regimen Choice

The full cohort included 733 patients (**Table 1**). Before PSM, patients receiving DL tended to be male, have grade 1 tumours, have pathological lymph node metastases and undergo more than 3–4 cycles of AC. The increasing practice patterns of DL changed over time; DL use increased from 68 patients in 2013 to 322 in 2016 (**Figure 2C**). There were 276 patients receiving DL as AC. However, twenty-three patients did not have any safety records of AC or were lost to follow-up. Finally, two hundred and fifty-three patients receiving DL were included in the PSM and followed with safety and survival analyses. The characteristics of 458 patients were similar between the two groups after PSM. Details regarding the distribution of patients treated with DL, DC and other chemotherapy regimens are shown in **Table 1** and **Figures 2A, B**.

**Table 1:** Characteristics of full and propensity score–matched cohorts.

Characteristics	Full Cohort			Propensity Score Matched		
	DL (n = 253)	DC (n = 480)	P	DL (n = 229)	DC (n = 229)	P
Sex			0.0069			1.000
Male	217 (85.77)	370 (77.08)		198 (86.46)	199 (86.9)	
Female	36 (14.23)	110 (22.92)		31 (13.54)	30 (13.1)	
Age (years)			0.949			1.000
<60	130 (51.38)	244 (50.83)		117 (51.09)	118 (51.53)	
>=60	123 (48.62)	236 (49.17)		112 (48.91)	111 (48.47)	
BMI			0.6533			0.948
<18.5	23 (9.09)	44 (10.67)		22 (9.61)	24 (10.48)	
18.5~24	194 (76.67)	356 (74.17)		174 (75.98)	171 (74.67)	
>=24	33 (13.04)	75 (15.63)		31 (13.54)	31 (13.54)	
Missing	3 (1.19)	5 (1.04)		2 (0.87)	3 (1.31)	
Smoking			0.093			0.179
Never	37 (14.62)	106 (22.08)		39 (17.03)	43 (18.78)	
Ever/current	201 (79.44)	335 (69.79)		176 (76.85)	167 (72.93)	
Missing	15 (5.93)	39 (8.13)		14 (6.12)	19 (8.29)	
Alcohol			0.599			0.273
Never	115 (23.96)	70 (27.67)		70 (30.57)	57 (24.89)	
Ever/current	336 (70.00)	172 (67.98)		148 (64.63)	163 (71.18)	
Missing	29 (6.04)	11 (4.35)		11 (4.80)	9 (3.93)	
Clinical stage			0.4642			0.500
Stage II	159 (62.85)	316 (65.83)		146 (63.76)	138 (60.26)	
Stage III	94 (37.15)	164 (34.17)		83 (36.24)	91 (39.74)	
Location of tumour			0.6268			0.336
Upper thoracic	45 (17.79)	72 (15.0)		36 (15.72)	36 (15.72)	
Middle thoracic	140 (55.34)	257 (53.54)		134 (58.52)	114 (49.78)	
Lower thoracic	60 (23.72)	122 (25.42)		55 (24.02)	65 (28.38)	
Missing	8 (3.16)	29 (6.04)		4 (1.75)	14 (6.11)	
Thickness of tumour			0.7347			0.589
<3 m	37 (14.62)	63 (13.13)		35 (15.29)	29 (12.67)	
>=3 cm	207 (81.82)	384 (80)		189 (82.53)	186 (81.22)	
Missing	9 (3.56)	33 (6.88)		5 (2.18)	14 (6.11)	
Histological grade			0.0399			0.715
Well differentiated (G1)	40 (15.81)	44 (9.17)		40 (17.47)	37 (16.16)	
Moderately differentiated (G2)	157 (62.06)	315 (65.63)		157 (68.56)	165 (72.05)	
Poorly differentiated (G3)	32 (12.65)	65 (13.54)		32 (13.97)	27 (11.79)	
Missing	24 (9.49)	56 (11.67)		0	0	
Lymphocyte infiltration			0.0164			0.844
No	162 (64.03)	319 (66.46)		149 (65.07)	152 (66.38)	
Yes	83 (32.81)	107 (22.29)		80 (34.93)	77 (33.62)	
Missing	8 (3.16)	54 (11.25)		0	0	
Cycles of AC			0.0021			0.933
1~4	231 (91.3)	396 (82.5)		210 (91.7)	208 (90.83)	
5~8	21 (8.30)	72 (15.0)		18 (7.86)	20 (8.73)	
>8	1 (0.40)	12 (2.5)		1 (0.44)	1 (0.44)	

### Toxicity of Therapy

After matching, leukopenia, neutropenia, nephrotoxicity and gastrointestinal symptoms were more frequent in the DC group. There were no significant differences in haemoglobin levels, platelet counts or hepatotoxicity between the two groups. For the ECG test, significantly more abnormal reports were recorded in the DC group. The details are provided in **Table 2**.

**Table 2:** Side effects of adjuvant therapy in the matched full cohort.

Toxicity	DL (n = 229)	DC (n = 229)	P
Leukopenia			0.0176
0	136 (59.39)	105 (45.85)	
I-II	79 (34.5)	99 (43.23)	
III-IV	6 (2.62)	12 (5.24)	
Missing	8 (3.49)	13 (5.68)	
Haemoglobin decreased			0.4042
0	112 (48.91)	124 (54.15)	
I-II	95 (41.49)	82 (35.81)	
III-IV	14 (6.11)	11 (4.8)	
Missing	8 (3.49)	12 (5.24)	
Thrombocytopenia			0.0600
0	95 (41.49)	112 (48.91)	
I-II	103 (44.98)	98 (42.79)	
III-IV	20 (8.73)	9 (3.93)	
Missing	11 (4.80)	10 (4.37)	
Neutropenia			0.0015
0	117 (51.09)	83 (36.24)	
I-II	86 (37.56)	122 (53.28)	
III-IV	15 (6.55)	10 (4.37)	
Missing	11 (4.80)	14 (6.11)	
Hepatotoxicity			0.3687
0	160 (69.87)	177 (77.29)	
I-II	40 (17.47)	34 (14.85)	
Missing	29 (12.66)	18 (7.86)	
Nephrotoxicity			<0.001
0	176 (76.86)	151 (65.94)	
I-II	32 (13.97)	61 (26.64)	
Missing	21 (9.17)	17 (7.42)	
Gastrointestinal symptoms			0.0011
0	74 (32.31)	46 (20.88)	
I-II	125 (54.59)	149 (65.07)	
III-IV	5 (2.18)	15 (6.55)	
Missing	25 (10.92)	19 (8.30)	
ECG			0.0068
Normal	135 (58.95)	111 (48.47)	
Abnormal	68 (29.69)	98 (42.79)	
Missing	26 (11.35)	20 (8.74)	

ECG, Electrocardiograph.

In the subgroup analysis of 1–2 cycles *versus* more than 2 cycles of DL, there was significantly less toxicity in the DL group for gastrointestinal symptoms (P=0.047) (**Table 3**). In the subgroup analysis of 1–2 cycles of AC, there was significantly less toxicity in the DL group for neutropenia (P=0.0113), ECG test reports (P=0.0052), nephrotoxicity (P=0.0031) and gastrointestinal symptoms (P=0.0018). The other factors were not different (**Table 4**). In the subgroup analysis of three to four cycles of AC, there was significantly less toxicity in the DL group for neutropenia (P=0.028) (**Table 5**).

**Table 3:** Subgroup analysis of side effects of adjuvant therapy (DL ≥ 2 cycles *versus* DL < 2 cycles).

Toxicity	DL < 2 Cycles (n = 118)	DL ≥ 2 Cycles (n = 111)	P
Leukopenia			0.7546
0	70 (59.32)	66 (59.46)	
I-II	39 (33.05)	40 (36.04)	
III-IV	4 (3.39)	2 (1.8)	
Missing	5 (4.24)	3 (2.7)	
Haemoglobin decreased			0.5342
0	53 (44.92)	59 (53.15)	
I-II	52 (44.07)	43 (38.74)	
III-IV	8 (6.78)	6 (5.41)	
Missing	5 (4.23)	3 (2.70)	
Thrombocytopenia			0.2884
0	55 (46.61)	40 (36.04)	
I-II	49 (41.53)	54 (48.65)	
III-IV	9 (7.63)	11 (9.91)	
Missing	5 (4.23)	6 (5.4)	
Neutropenia			0.186
0	61 (51.69)	56 (50.45)	
I-II	41 (34.75)	45 (40.55)	
III-IV	11 (9.32)	4 (3.6)	
Missing	5 (4.24)	6 (5.4)	
Liver disorder			0.1104
0	89 (75.42)	71 (63.96)	
I-II	16 (13.56)	24 (21.62)	
Missing	13 (11.02)	16 (14.42)	
Renal disorder			0.2485
0	94 (79.66)	82 (73.87)	
I-II	13 (11.02)	19 (17.12)	
Missing	11 (9.32)	10 (9.01)	
Gastrointestinal symptoms			0.047
0	46 (38.98)	28 (25.23)	
I-II	56 (47.46)	69 (62.16)	
III-IV	3 (2.54)	2 (1.8)	
Missing	13 (11.02)	12 (10.81)	
ECG			0.1053
Normal	74 (62.71)	61 (54.95)	
Abnormal	29 (24.58)	39 (35.14)	
Missing	15 (12.71)	11 (9.91)	

ECG, Electrocardiograph.

**Table 4:** Subgroup analysis of side effects of 1–2 cycles of adjuvant therapy (DL *versus* DC).

Toxicity	DL (n = 118)	DC (n = 90)	P
Leukopenia			0.0664
0	70 (59.32)	38 (42.22)	
I-II	39 (33.05)	40 (44.44)	
III-IV	4 (3.39)	5 (5.56)	
Missing	5 (4.24)	7 (7.78)	
Haemoglobin decreased			0.7341
0	53 (44.91)	46 (51.11)	
I-II	52 (44.07)	36 (40.00)	
III-IV	8 (6.78)	6 (6.67)	
Missing	5 (4.24)	2 (2.22)	
Thrombocytopenia			0.5729
0	55 (46.60)	42 (46.67)	
I-II	49 (41.53)	42 (46.67)	
III-IV	9 (7.63)	4 (4.44)	
Missing	5 (4.24)	2 (2.22)	
Neutropenia			0.0113
0	61 (51.69)	29 (32.22)	
I-II	41 (34.75)	47 (52.22)	
III-IV	11 (9.32)	5 (5.56)	
Missing	5 (4.24)	9 (10.00)	
Liver disorder			0.5598
0	89 (75.42)	68 (75.56)	
I-II	16 (13.56)	16 (17.77)	
Missing	13 (11.02)	6 (6.67)	
Renal disorder			0.0031
0	94 (79.66)	58 (64.44)	
I-II	13 (11.02)	25 (27.78)	
Missing	11 (9.32)	7 (7.78)	
Gastrointestinal symptoms			0.0018
0	46 (38.98)	18 (20.00)	
I-II	56 (47.46)	57 (63.33)	
III-IV	3 (2.54)	8 (8.89)	
Missing	13 (11.02)	7 (7.78)	
ECG			0.0052
Normal	74 (62.71)	40 (44.45)	
Abnormal	29 (24.58)	39 (43.33)	
Missing	15 (12.71)	11 (12.22)	

ECG, Electrocardiograph.

**Table 5:** Subgroup analysis of side effects of 3–4 cycles of adjuvant therapy (DL *versus* DC).

Toxicity	DL (n = 92)	DC (n = 118)	P
Leukopenia			0.5475
0	54 (58.7)	61 (51.69)	
I-II	33 (35.87)	46 (38.98)	
III-IV	2 (2.17)	5 (4.25)	
Missing	3 (3.26)	6 (5.08)	
Haemoglobin decreased			0.7844
0	49 (53.26)	65 (55.08)	
I-II	36 (39.13)	40 (33.9)	
III-IV	5 (5.43)	5 (4.24)	
Missing	2 (2.18)	8 (6.78)	
Thrombocytopenia			0.1135
0	36 (39.13)	57 (48.31)	
I-II	41 (44.57)	48 (40.68)	
III-IV	10 (10.87)	5 (4.24)	
Missing	5 (5.43)	8 (6.77)	
Neutropenia			0.028
0	51 (55.43)	47 (39.83)	
I-II	34 (36.96)	64 (54.24)	
III-IV	1 (1.09)	3 (2.54)	
Missing	6 (6.52)	4 (3.39)	
Liver disorder			0.2567
0	62 (67.39)	93 (78.81)	
I-II	18 (19.57)	17 (14.41)	
Missing	12 (13.04)	8 (6.78)	
Renal disorder			0.1254
0	70 (76.09)	80 (67.80)	
I-II	15 (16.30)	31 (26.27)	
Missing	7 (7.61)	7 (5.93)	
Gastrointestinal symptoms			0.1657
0	24 (26.09)	25 (21.19)	
I-II	58 (63.04)	75 (63.56)	
III-IV	1 (1.09)	7 (5.93)	
Missing	9 (9.78)	11 (9.32)	
ECG			0.7674
Normal	49 (53.26)	62 (52.54)	
Abnormal	33 (35.87)	47 (39.83)	
Missing	10 (10.87)	9 (7.63)	

ECG, Electrocardiograph.

### Survival

**Figure 3:**
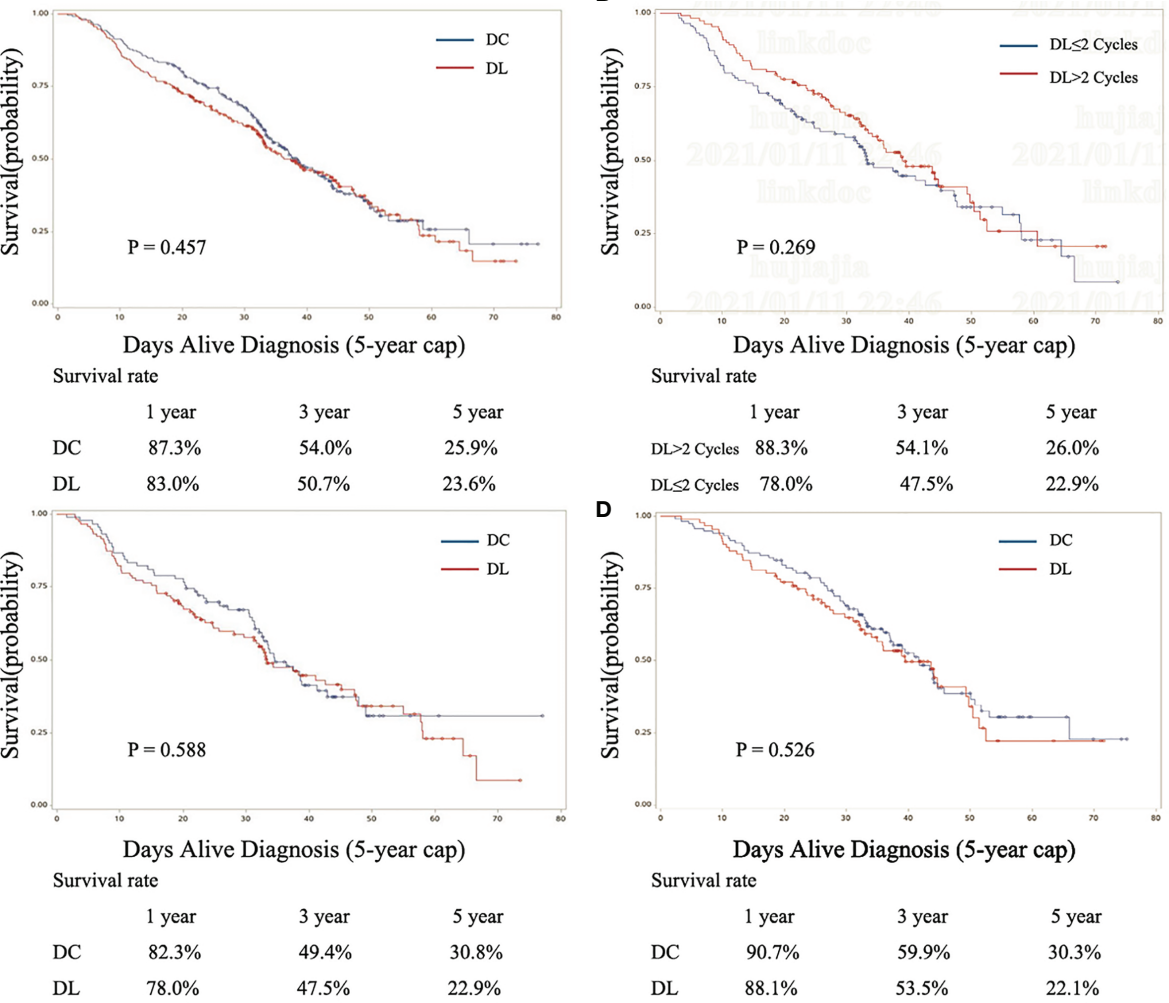
Kaplan-Meier curves for the 3-year survival outcomes of propensity score–matched patients by regimen (DL and DC). **(A)** Fully matched cohort, **(B)** DL subgroup, **(C)** 1–2 cycles of AC group of DL and DC, and **(D)** 3–4 cycles of AC group of DL and DC. DL, docetaxel+ lobaplatin; DC, docetaxel+ cisplatin.

In the matched subset, the follow-up was conducted from 1.6 to 77.0 months. The mean follow-up period was 31.2 months. The median follow-up period was 31.1 months in the DL group and 32.9 months in the DC group. In the matched subset, 23.6% of DL users and 25.9% of DC users were alive at 5 years, log-rank test P=0.457 (median survival time, DL 36.2 months, 95% CI, 32.8 to 44.6; DC 38.4 months, 95% CI, 33.9–43.4; **Figure 3A**). In the first year, the DC group had a slightly higher OS (87.3%) than the DL group (83.0%); the same situation was observed for the 3-year OS (DC 54.0% and DL 50.7%). There were no statistically significant differences (**Figure 3A**). In the subgroup analysis of 1–2 cycles *versus* more than 2 cycles of DL, the five-year OS was 22.9% *versus* 26.0% (P=0.269; **Figure 3B**). In the subgroup analysis of 1–2 cycles of AC, the 5-year OS was 22.9% for DL users and 30.8% for DC users (P=0.588; **Figure 3C**). In the subgroup analysis of 3–4 cycles of AC, the 5-year OS was 22.1% for DL users and 30.3% for DC users (P=0.526; **Figure 3D**).

## Discussion

In this national project of AC after radical resection for ESCC, among matched patients, we found no significant differences in the 5-year OS between DL and DC users. However, according to WHO-based chemotherapy-related toxicities, DC users had significantly worse leukopenia, nephrotoxicity and gastrointestinal symptoms. Regarding the ECG test, DC users also had significantly more abnormal reports. In the DL subgroup, patients receiving more than 2 cycles of DL had the same survival benefits as those receiving 1–2 cycles of DL. The difference in toxicity between the two groups involved worse gastrointestinal symptoms in the DL group with more than 2 cycles.

A study showed no significant difference in the survival of LBP-based regimens *versus* cisplatin for metastatic breast cancer (10), which is consistent with our results. Although survival with cisplatin was slightly high, there was still no significant difference in this large sample size cohort. In the DL subgroup analysis, we found that the survival of patients treated with more than two cycles was perhaps better than that of patients treated with 1–2 cycles of AC, without statistical significance, while more cycles of DL increased gastrointestinal symptoms. Four cycles of AC are the standard treatment for lung cancer (11). Four cycles were better than 2 cycles (11). However, since retrospective data were used, the results should be interpreted with caution. Patients with poor physical condition may be less able to complete 3–4 cycles of AC. Patients with different chemotherapy cycles may be screened. Patients with multiple cycles may have better physical, financial and family support. These data are not within the range of our analysis. Therefore, the same survival benefit of different chemotherapy cycles cannot be fully interpreted, as the 2 cycles were sufficient. Randomized controlled clinical trials are still needed to identify and exclude potential confounders.

The lower toxicity of LBP has been demonstrated in breast cancer (12), lung cancer (13), oesophageal carcinoma (14), and hepatic cancer (15). Patients with ESCC after surgery usually have worse PSs than those with other types of cancer. Previous research has reported the use of LBP in ESCC (16), but no study has compared the effectiveness and safety of DL and DC in the AC setting of ESCC. Lower toxicity is very important for patients to complete 4 cycles of AC. LBP, a third-generation platinum anticancer drug developed by the German company ASTA, has been reported in the international literature to have limited nephrotoxicity without the need to perform hydration during chemotherapy (3). The major dose-limiting toxicity of LBP was thrombocytopenia in a past report (3, 17, 18). Similarly, the most frequent grade 3–4 toxicity in our study was thrombocytopenia (8.73%). However, compared with the DC group, the toxicity of thrombocytopenia in the DL group was not different (P=0.060) in our data. Overall, the grade 3–4 toxicity of LBP was less than 10%, which was acceptable. No grade 3 or 4 hepatotoxicity or nephrotoxicity was observed. Renal toxicity and cardiac dysfunction may be reduced. Compared with DC, DL had much lower incidences of nephrotoxicity and fewer abnormal ECG reports during AC. Some previous reports of LBP showed neutropenia (17, 19). However, in our study, the incidence of neutropenia with LBP was much lower than that with cisplatin. AC with cisplatin brings high pressure to patients because of its toxicity (20). It can easily induce drug resistance (20). LBP has no crossing drug resistance with other platinum-based drugs (3).

In the year-by-year OS results of DC *versus* DL, the DC group consistently had better survival rates but without statistical significance. The toxicity of DC was much worse than that of DL. In a previous report, compared with cisplatin, LBP had lower toxicity, lower physical and mental pressure, and less stimulation of the vasculature (21). To control and reduce the toxicity of AC in certain patients after oesophagectomy, DL may be a good choice since it has the same survival outcomes with less toxicity.

Several limitations that are common to observational analyses could be found in this study. All toxicity data were collected in the inpatient department. The full extent of toxicities, especially after discharge, could not be collected. Second, there was no information on recurrence. Thus, it was impossible to analyse disease-free survival (DFS). Similarly, this retrospective observational study did not collect the cause of death. We were unable to calculate the tumour-related OS. Although propensity score adjustment was used, the unmeasured factors may still have remained confounding factors. Although the multicentre study had a large sample size, the standardization of all data was much more difficult than that for a single centre study. Finally, for the evaluation of the toxicity of AC, the Common Terminology Criteria for Adverse Events (CTCAE) was not adopted.

There was no information available to clinicians choosing between cisplatin and LBP in the adjuvant setting for ESCC. Some studies have demonstrated the safety and effectiveness of LBP in advanced ESCC. No head-to-head clinical trial has compared DC and DL in terms of efficacy or toxicity. Prospective randomized studies are unlikely to be conducted in the near future. The large population from multiple centres and rigorous retrospective studies can inform clinical care by offering information about the outcomes of different treatments. The results of this study can fill a crucial knowledge gap.

In conclusion, DL has the same long-term survival benefit and lower chemotherapy-related toxicity than DC as AC in the treatment of ESCC. However, the data included in this retrospective study come from different research centres. It is inevitable that some data are missing, which made it impossible to evaluate the DFS of patients. The results need to be confirmed by large prospective controlled studies.

## Author’s Note

The abstract was accepted as an oral presentation at the 26th European Conference on General Thoracic Surgery at Ljubljana, Slovenia, in May 2018.

## Data Availability Statement

The data analyzed in this study is subject to the following licenses/restrictions: The data was in LinkDoc company. The access of the dataset should be approved by ethics review committee of all the included multiple centers. Requests to access these datasets should be directed to dingjing201305@163.com.

## Ethics Statement

This study was approved by the ethics review committee of the Affiliated Cancer Hospital of ZhengZhou University/Henan Cancer Hospital approved officially with the approval number 2017405. The patients/participants provided their written informed consent to participate in this study.

## Author Contributions

YL and YZ designed the study. XL, HS, JH, and LL performed the experiments. JH and LL analysed the data. ZY and GL wrote the manuscript. All authors contributed to the article and approved the submitted version.

## Funding

This work was supported by the National Natural Science Foundation of China, NSFC (grant number 82002521); Natural Science Foundation of Henan Province (grant number 202300410389), China; Henan Anti-Cancer Association Youth Talent Project (grant number 2019HYTP018, 2019), China; Wu Jieping Medical Foundation (CN) (grant number 320.6750.2020-15-1), China, Special Program for Basic Resource Survey of the Ministry of Science and Technology (grant number 2019FY101101).

## Conflict of Interest

Authors JH and LL were employed by LinkDoc Technology Co., Ltd.

The remaining authors declare that the research was conducted in the absence of any commercial or financial relationships that could be construed as a potential conflict of interest.

## Publisher’s Note

All claims expressed in this article are solely those of the authors and do not necessarily represent those of their affiliated organizations, or those of the publisher, the editors and the reviewers. Any product that may be evaluated in this article, or claim that may be made by its manufacturer, is not guaranteed or endorsed by the publisher.

## Abbreviations and Acronyms

LBP, lobaplatin; ESCC, oesophageal squamous cell carcinoma; OS, overall survival; PSM, propensity score matching; AC, adjuvant chemotherapy; PSs, performance scores; BMI, body mass index; DL, docetaxel+LBP; DC, docetaxel+ cisplatin; WHO, World Health Organization; ECG, electrocardiogram.
